# A new species of *Lithobius* (Monotarsobius) Verhoeff, 1905 (Lithobiomorpha, Lithobiidae) from China

**DOI:** 10.3897/zookeys.82.823

**Published:** 2011-02-23

**Authors:** Sujian Pei, Huiqin Ma, Baojun Shi, Dayong Wu, Wenjie Zhou

**Affiliations:** 1 Department of Life Sciences, Hengshui University, Hengshui, Hebei 053000, P. R. China; 2 Scientific Research Office, Hengshui University, Hengshui, Hebei 053000, P. R. China

**Keywords:** new species, *Lithobius (Monotarsobius) songi* sp. n., Lithobiidae, Hebei, China

## Abstract

The present paper deals with a new species of the genus Lithobius Leach, 1814, Lithobius (Monotarsobius) songi sp. n.(Lithobiomorpha: Lithobiidae) recently discovered in Hebei Province, China. Morphologically it resembles Lithobius (Monotarsobius) holstii (Pocock, 1895) from China and Japan but could be well distinguished from latter by havinga Tömösváry’s organ slightly smaller than the adjoining ocelli, different leg plectrotaxy and tridentate claw of female gonopods. A key to the Chinese Lithobius (Monotarsobius)species is presented.

## Introduction

The subgenus Monotarsobius Verhoeff, 1905 of genus Lithobius Leach, 1814 is characterized by the presence of fused tarsi of legs 1–13 and antennal articles fixed at 20 or thereabouts (Eason 1992). Presently, this subgenus comprises one hundred species known from Asia, Europe, and North Africa (Zapparoli 2006). Very little attention has been paid to the study of Lithobiomorpha in China, with only 15 genera and 66 species having been recorded up to now (e.g. Takakuwa and Takashima 1949; Wang 1959, 1963; Zhang 1996; Ma et al. 2007a, b, 2008a, b). As regards the investigation of Lithobius (Monotarsobius) only 8 species have hitherto been recorded (cf. Trotzina 1895; Pocock 1895; Takakuwa 1940, 1941, 1942; Zalesskaja 1978; Wang and Mauriès 1996; Eason 1997; Chao 2005; Ma et al. 2009a, b). Takakuwa (1941) described Lithobius (Monotarsobius) obtusus and Lithobius (Monotarsobius) ramulosus from Taiwan and later on (Takakuwa 1940, 1942) recorded also Lithobius (Monotarsobius) crassipes and Lithobius (Monotarsobius) holstii from the island. Zalesskaja (1978) recorded two other species, Lithobius (Monotarsobius) alticus and Lithobius (Monotarsobius) crassus from Xinjiang Autonomous Region. Eason (1997) recorded Lithobius (Monotarsobius) ferganensis from Xinjiang Autonomous Region, while Ma et al. (2009b) described Lithobius (Monotarsobius) subspinipes from Hainan and Hebei provinces. After a study of recently collected material from Hebei Province, we came across a new species of Lithobius (Monotarsobius), which is described below.

## Methods

All specimens were hand-collected under stones. The material was examined with the aid of a Motic-C microscope, made in China. Colour description is based on specimens in 75% ethanol, and body length is measured from anterior margin of the cephalic plate to posterior end of telson. Type specimens are preserved in 75% ethanol and deposited in the College of Life Sciences, Hebei University, Baoding, China; some nontype material is deposited in the Department of Life Sciences, Hengshgui University, Hengshui, China. Terminology for external anatomy follows Bonato et al.(2010).

The following abbreviations are used in the text and tables: T, TT = tergite, tergites; S, SS = sternite, sternites; C = coxa, t = trochanter, P = prefemur, F = femur, T = tibia, a = anterior, m = median, p = posterior.

## Taxonomic part

**Lithobiidae Newport, 1844**

### Lithobius (Monotarsobius) songi sp. n.

urn:lsid:zoobank.org:act:9739FCCD-35E8-477E-AA81-22EA6B83F40A

Figs 1–6 

#### Etymology:

The specific name is a patronym in honor of the zoologist Dr. Daxiang Song, Academician at the Chinese Academy of Sciences.

#### Material examined:

**Figures 1–6.**
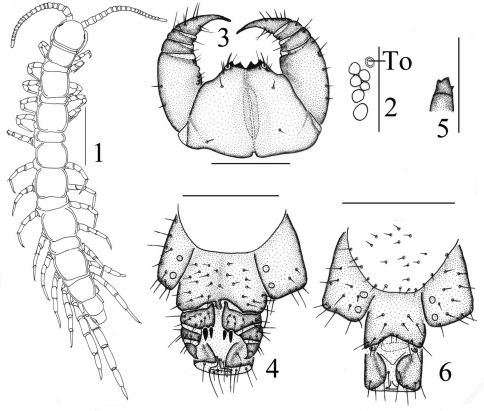
Lithobius (Monotarsobius) songi sp. n., **1–5** holotype, female: **1** habitus, dorsal view, scale 1 mm **2** ocelli andTömösváry’s organ (To),lateral view, scale 250 µm **3** forcipular coxosternite, ventral view, scale 500 µm **4** posterior segments and gonopods, ventral view, scale 500 µm **5** claw of gonopods, internal view, scale 250 µm **6** paratype, male: gonopods, ventral view, scale 250 µm.

**Holotype:** female (Fig. 1), body length 6.9 mm, cephalic plate length 0.7 mm, breadth 0.7 mm; from Qingliangdian Town, Wuyi County, Hengshui City, Hebei Province, 37°06'N, 115°08'E, 35 m, 6 May 2005, leg. H. Ma. **Paratypes:** 2 ♀♀, 1 ♂, same data as holotype.

#### Other material:

1 ♀♀, 3 ♂♂, Xiaowutai National Natural Reserve, Yu County, Zhangjiakou City, Hebei Province, 39°54'N, 115°00'E, 1236 m, 21 August 2005, leg. Z. Zhang and H. Ma.

#### Diagnosis:

A Lithobius (Monotarsobius) species with body length 5.9–6.9 mm, antennae composed of 19–21 articles, commonly 19+19; 6–7 ocelli on each side, commonly 6, arranged in 2 irregular rows; Tömösváry’s organ moderately small, slightly smaller than adjoining ocelli; 2+2 coxosternal teeth; porodonts moderately slender and long, posterolateral to lateral tooth; posterior angles of all tergites without triangular projections; coxal pores 1222, oval to round; female gonopods with 2+2 moderately large, coniform spurs; terminal claw tridentate; male gonopods short and small, with 1–2 long setae on terminal segment.

#### Description:

Body 5.9–6.9 mm long; cephalic plate 0.6–0.7 mm long, 0.6–0.7 mm wide. Colour: tergites and basal articles pale brown; transition to yellow brownish from the seventh or eighth articles onwards, the terminal article yellow brown; tergites pale chestnut; pleural region pale gray; SS pale orange; distal part of forcipules brown, the remaining part of forcipules, forcipular coxosternite and SS 14 and 15 pale yellow-brownish; all legs pale yellow-brownish, tarsi of all legs yellow-brown.

Antennae composed of 19+19–21+21 articles (Fig. 1), most often 19+19; basal article almost as long as wide, second one markedly longer than wide, succeeding articles gradually shortening; terminal article typically longer than wide, up to 2.3–2.9 times longer than wide. Antennal setation: abundant setae on antennal surface, but fewer setae on outer side and ventral and dorsal side in basal articles, gradual increase in density of setae to about fourth or fifth article, then more or less constant.

Cephalic plate smooth, convex, as long as broad, covered with sparse tiny setae; anterior part of the cephalic capsule with shallow median sulcus; pigment concentrated as close netlike veins, few short to long setae scattered along the marginal ridge; lateral marginal ridge continuous; posterior margin straight, without widening in middle part (Fig. 1).

Six–seven ocelli on each side of cephalic plate (Fig. 2), more often 6, arranged in 2 irregular rows; the terminal one larger, the ocelli near the dorsal slightly larger, the ocelli near the ventral slightly smaller; overhanging the lateral margin of the cephalic plate; ocelli gently bulging, translucent, usually darkly pigmented.

Tömösváry’s organ moderately small (Fig. 2–To), nearly rounded, situated ventrad to anterolateral margin of cephalic pleurite, slightly smaller than the adjoining ocelli.

Coxosternite (Fig. 3) approximately trapezoidal, anterior margin moderately narrow with 2+2 comparatively sharp coxosternal teeth, median diastema relatively deep, V-shaped (Fig. 3); coxosternal shoulder lacking; porodonts lying posterolateral to the lateral tooth, comparely long and slender, without a bulge at the base. Moderately short to long setae sparsely scattered over the dental margin, comparatively long and thick near the dental margin.

All tergites moderately smooth, without wrinkles, backside slightly hunched, tiny setae scattered very sparsely over the surface; T 1 generally subrectangular, anteriorly broadened; T 1 slightly narrower than T 3 and the cephalic plate, the latter slightly wider than T3; lateral marginations of all tergites continuous, setae scattered sparsely along the lateral borders, more setae on anterior angles of tergites; posterior margin of TT 1, 3, 5, 7, 8, 10, 12 and 14 slightly concave; all tergites without posterior triangular projections (Fig. 1).

All sternites posterolaterally narrower than anterolaterally, generally trapeziform, moderately smooth, 2–4 moderately setae on anterior part of each sternite, 2–3 longer setae on posterior part of each sternite.

Legs strong, tarsus 1–2 articulation fused on legs 1–13, well-defined on legs 14 and 15; claw moderately long and curved ventrad in all legs; accessory spur on both anterior and posterior side of claw of legs 1–14, anterior accessory spur moderately long and thicker, forming a moderately large angle with the claw; posterior accessory spur short and slender, forming a comparatively small angle with the claw; no accessory claws on leg 15; short to moderately long setae scattered over the surface of legs 1–13, tarsi generally more setose, few setae on legs 14–15; legs 14 and 15 markedly thickened,  the male more thicken than the female, tarsus 1 about 4.0–5.3 times longer than wide, tarsus 2 about 67%–81% length of tarsus on legs 15. Legs’ plectrotaxy: as in Table 1.

Coxal pores arranged in a row, ovate to round, moderately small, 1222. Pore-field set in a slightly shallow groove, 8 short to moderately long setae scattered sparsely over the margin of shallow groove.

Female S 15 posterolaterally narrower than anterolaterally, generally trapeziform, straight posteromedially; short to long setae scattered very sparsely over its surface and lateral margins. The sternite of genital segment usually well sclerotised, wider than long, posterior border moderately deeply concave between condyles of gonopods, except for a small, median approximately rhombic bulge, distally lightly sclerotised; short to moderately long setae evenly scattered over the surface of genital sternite except for middle and anterior parts. Female gonopods: basal article moderately broad, bearing 8 moderately long setae, arranged in 3 irregular rows, and 2+2 small coniform spurs; inner spur slightly smaller and more anterior than the outer (Fig. 4); second article with 6 moderately long setae, arranged in 2 irregular rows; usually 3–4 moderately long setae on the surface of third article; terminal claw tridentate (Fig. 5), dorsal and ventral tooth about same in size.

Male S 15 posterolaterally narrower than anterolaterally, generally trapeziform, straight posteromedially; short to long setae scattered very sparsely over its surface and lateral margins. The sternite of genital segment usually well sclerotised, wider than long; comparatively long setae about evenly scattered on the ventral surface, slightly fewer near S15. Posterior margin of the sternite of the genital segment quite deeply concave between gonopods, no bulge medially; gonopods short and small, only a small hemispherical bulge, with 1–2 long setae on surface, terminal slightly sclerotised (Fig. 6).

#### Distribution:

Known only fromthe Hebei Province (Hengshui and Zhangjiakou Cities), NE China.

**Table 1.** Legs’ plectrotaxy of Lithobius (Monotarsobius) songi sp. n. Letters in brackets indicate variable spines.

legs	ventral					dorsal				
		C	t	P	F	T	C	t	P	F	T
1			p	a	m			p	ap	a
2			p	am	m			p	ap	ap
3–9				am	m				ap	ap
10				am	m				p	ap
11			(m)	am	m			p	p	p
12			mp	am	am			p	p	p
13		(m)	mp	(a)m(p)	m			mp	p	p
14		m	mp	m		m		mp		
15		m	mp	m		m		mp		

#### Remarks:

Havingan eye composed of 6–7 ocelli and about 20 antennal articles, the new species resembles Lithobius (Monotarsobius) holstii (Pocock, 1895) from China and Japan (Takakuwa 1941). However, it is well distinguished from the latter by having Tömösváry’s organ slightly smaller than the adjoining ocelli, different leg plectrotaxy and tridentate claw of female gonopods (bidentate in Lithobius (Monotarsobius) holstii). It differs from Lithobius (Monotarsobius) subspinipes Ma et al., 2009 by having smaller Tömösváry’s organ, different leg plectrotaxy and moderaterly setose legs (vs. only sparse setae in Lithobius (Monotarsobius) subspinipes).

#### Habitat preferences:

The type series has been collected in a roadside of a mountain pine tree forest and under Chinese jujube trees in champaign environments.

#### Key to the Chinese species of Lithobius (Monotarsobius)

To assist in the identification of the Chinese species of Lithobius (Monotarsobius), the following key is offered. This key emphasizes characters that can be examined without much dissection or high-magnification microscopy; moreover, these characters are specific to the taxa occurring in China.

**Table:** 

1	Four ocelli on each side of cephalic plate, 17+17 antennal articles	Lithobius (Monotarsobius) crassus (Loksa, 1965)
–	Five or more ocelli on each side of cephalic plate, not less than 18+18 antennal articles	2
2	Five ocelli on each side of cephalic plate	Lithobius (Monotarsobius) alticus (Loksa, 1965)
–	Six or more ocelli on each side of cephalic plate	3
3	With spines on the second article of female gonopod	4
–	Without spines on the second article of female gonopod	5
4	With two spines on the second article of female gonopod, six–ten ocelli on each side of cephalic plate, 1222–2222 coxal pores	Lithobius (Monotarsobius) ferganensis (Trotzina, 1894)
–	With three spines on the second article of female gonopod, eight–eleven ocelli on each side of cephalic plate, 2222–3443 coxal pores	Lithobius (Monotarsobius) crassipes L. Koch, 1862
5	Terminal claw of female gonopod simple	Lithobius (Monotarsobius) ramulosus (Takakuwa, 1941)
–	Terminal claw of female gonopod bidentate or tridentate	6
6	Terminal claw of female gonopod tridentate	7
–	Terminal claw of female gonopod bidentate	8
7	Tomosvary’s organ slightly smaller than adjoining ocellus; terminal ocellus largest	Lithobius (Monotarsobius) songi sp. n.
–	Tomosvary’s organ slightly larger than adjoining ocellus or about same in size; terminal two ocelli largest	Lithobius (Monotarsobius) subspinipes Ma et al., 2009
8	Tomosvary’s organ larger than largest ocellus, antennae 20–25 articles	Lithobius (Monotarsobius) holstii (Pocock, 1895)
–	Tomosvary’s organ about same size as the adjoining ocelli, antennae 19 articles	Lithobius (Monotarsobius) obtusus (Takakuwa, 1941)

## Supplementary Material

XML Treatment for Lithobius (Monotarsobius) songi
